# Root Filling

**Published:** 1869-04

**Authors:** S. P. Cutler

**Affiliations:** Holly Springs, Miss.


### ROOT FILLING.

BY S. P. CUTLER, M. D., A. E. G., D. D. S., HOLLY SPRINGS, MISS.

I often use Richardson's Styptic Colloid rather consistent. Saturate a small wad of cotton, rolled hard, of suitable size ; introduce into cavity of root, and press up and condense thoroughly; after waiting a few moments for evaporation, introduce another wad and condense as before, and continue until filled to the desired point; after waiting a few minutes for evaporation, the crown may be filled in the usual way. The advantages claimed are these: first, there is found an insoluble and impenetrable mass wholly indestructible, and should there be any animal matters at apex of root, a colloidate, styptate, tannate, or whatever it may be called, is formed, which becomes wholly unchangeable and is a non-conductor. The base is the same, minus the tannin of Rose Pearl, namely: lignin. The Colloid may be used to fill interstices around the teeth in gold plates.

